# Prevalence of Sodium and Fluid Restriction Recommendations for Patients with Pulmonary Hypertension

**DOI:** 10.3390/healthcare3030630

**Published:** 2015-07-28

**Authors:** Tonya Zeiger, Giovanna Cueva Cobo, Christine Dillingham, Charles D. Burger

**Affiliations:** Pulmonary and Critical Care Medicine, Mayo Clinic, 4500 San Pablo Rd, Jacksonville, FL 32224, USA; E-Mails: zeiger.tonya@mayo.edu (T.Z.); Cueva.giovanna@mayo.edu (G.C.C.); cdilling@wakehealth.edu (C.D.)

**Keywords:** sodium restriction, fluid restriction, pulmonary hypertension

## Abstract

*Background*: Patients with pulmonary hypertension (PH) are often afflicted with the consequences of right heart failure including volume overload. Counseling to assist the patient in the dietary restriction of sodium and fluid may be underutilized. *Methods*: Consecutive patients seen in the PH Clinic at Mayo Clinic in Florida from June to November 2013. *Results*: 100 patients were included; 70 were women and most had group 1 PH (*n* = 69). Patient characteristics using mean (±SD) were: Age 63 ± 13 years, functional class 3 ± 1, brain natriuretic peptide 302 ± 696 pg/mL, 6-min walk 337 ± 116 m, right atrial pressure 8 ± 5 mmHg, and mean pulmonary artery pressure 42 ± 13 mmHg. Overall, 79 had had complete (32) or partial instruction (47) and 21 had no prior counseling to restrict sodium or fluid. Of the 47 with partial instruction, 42 received complete education during the PH Clinic visit. Of the 21 without prior instruction, 19 received complete education during the PH visit. Seven patients with the opportunity to have their education enhanced or provided did not receive any additional counseling during the PH visit. *Conclusion*: Sodium and fluid restriction is an important but perhaps underutilized strategy to manage volume overload in patients with right heart failure. Focused questioning and education may permit an increase in the patients receiving instruction in this regard.

## 1. Introduction

Pulmonary arterial hypertension (PAH) is produced by pulmonary vascular narrowing that results in elevated pulmonary vascular resistance and right heart pressure [1]. Patients with PAH often are afflicted with the consequences of right heart failure including volume overload. Clinical signs and symptoms include worsening dyspnea and peripheral edema. Other types of pulmonary hypertension (PH) may have similar presentations. Clinicians often prescribe diuretics to control the fluid retention; however, counseling and education to assist the patient to also restrict the daily intake of sodium and fluid may be underutilized.

The combination of diuretic administration along with fluid and sodium restriction has been shown to reduce hospital admissions in recently compensated left heart failure [2]. Accordingly, dietary restriction has been incorporated into contemporary strategies to manage heart failure [3]. Education seems to reinforce the importance of home practice and improve compliance [4]. In addition, compliance with non-pharmacological strategies to manage heart failure seems to improve outcomes [5]. Little is published regarding the same approach with right heart failure due to PAH; however, the same benefit of diuretic therapy with sodium and fluid restriction likely exists [6]. In addition, such an approach is recommended in published guidelines for management of PAH [1,7].

The purpose of the study was simply to assess the frequency of counseling to reduce sodium and fluid intake and to explore whether specific targets were provided to all types of PH seen in the PH Center at Mayo Clinic in Jacksonville, Florida.

## 2. Methods

The study was approved by the Institutional Review Board. Adult patients with PH evaluated in the PH Center at Mayo Clinic in Jacksonville, Florida (MCF) were eligible. As part of the routine clinical assessment, the patients completed a brief questionnaire to determine if they had received counseling to restrict sodium and fluid from any prior healthcare providers. Consecutive patients from June through November 2013 were then reviewed to confirm and collect the questionnaire responses as well as the following information: World Health Organization (WHO) diagnostic group, functional class, brain natriuretic peptide (BNP) in picograms per milliliter (pg/mL), six-minute walk distance (6MWD) in meters (m), and echocardiogram measurement of both right atrial (RAP) and mean pulmonary artery pressure (MPAP) in mmHg. The patient’s clinical status was determined by the evaluating physician as stable, improved, or worsening. For purposes of this study, the patient is categorized as stable if either stable or improved and unstable if worsening. The presence or absence of peripheral edema was recorded. The presence and type of diuretic therapy was collected, but not the specific dose. Patients were classified according to predetermined groups: Group A if the patient had received counseling prior to the current visit to restrict sodium and/or fluid and group B if not. Subgroups were defined for both group A and B. Group A subgroups were as follows: Subgroup A1 if the patient had been previously directed to restrict both sodium and fluid including specific target amounts (e.g., number of mg sodium or ounces of fluid daily); A2 if the patient had been counseled to restrict only fluid or sodium but not both or was not given specific target amounts and then received instruction for both during the current PH Clinic visit (educational intervention provided); and A3 if the patient had been counseled to restrict only fluid or sodium but not both and then did not receive instruction for both during the current PH visit (*i.e.*, a missed educational intervention opportunity). Group B subgroups were as follows: B1 if the patient had not received counseling prior to the current visit to restrict sodium and/or fluid and then received instruction for both during the current PH Clinic visit (educational intervention provided); or B2 if the patient had not been counseled to restrict only fluid and/or sodium and then did not receive instruction for both during the current PH visit (*i.e.*, a missed educational intervention opportunity). In summary, subgroup A1 had previously received appropriate counseling for sodium and fluid restriction and therefore did not require further education (see Table 1). Subgroups A2 and B1 were patients requiring additional education and it was provided in the PH Clinic visit; however, subgroups A3 and B2, who also required supplemental counseling, did not receive it during the PH visit and represent an opportunity lost. Descriptive comparisons of the subgroups are provided.

**Table 1:** Description of groups and subgroups by classification of sodium and fluid restriction prior to and at the time of the Pulmonary Hypertension Clinic visit (*n* = 100).

Group/Subgroup	Definition	No. Patients
A	Prior counseling Na/fluid restriction YES	**79**
A1	Both Na/fluid restriction—No further education needed	32
A2	Only Na or fluid restriction (not both), PH Clinic education YES	42
A3	Only Na or fluid restriction (not both), PH Clinic education NO	5
B	Prior counseling Na/fluid restriction NO	**21**
B1	Prior counseling Na/fluid restriction NO, PH Clinic education YES	19
B2	Prior counseling Na/fluid restriction NO, PH Clinic education NO	2

Key: Na = sodium; No. = number; PH = pulmonary hypertension.

## 3. Results

Questionnaires and associated medical records were reviewed in 103 consecutive patients. Three patients had PH excluded as a diagnosis and therefore were not included in the analysis. Table 2 provides demographic and PH clinical information for the entire cohort (*n* = 100) as well as the subgroups. The entire cohort was mostly women with moderate to severe PH as assessed by functional class, BNP, 6MWD, and echocardiogram MPAP. Conversely, echocardiogram estimation of the RAP was only mildly elevated and the patients were clinically stable at the time of the outpatient evaluation. The type of PH as defined by WHO diagnostic groups was as follows: Group 1 PAH (*n* = 59); group 2 pulmonary venous hypertension (*n* = 30); group 3 PH in association with lung disease (*n* = 5); and group 4 chronic thromboembolic PH (*n* = 6). Nearly three-quarters (73%) were on diuretic therapy, most often a loop-inhibiting diuretic (e.g., furosemide) with or without an additional class diuretic (e.g., spironolactone).

**Figure 1:**
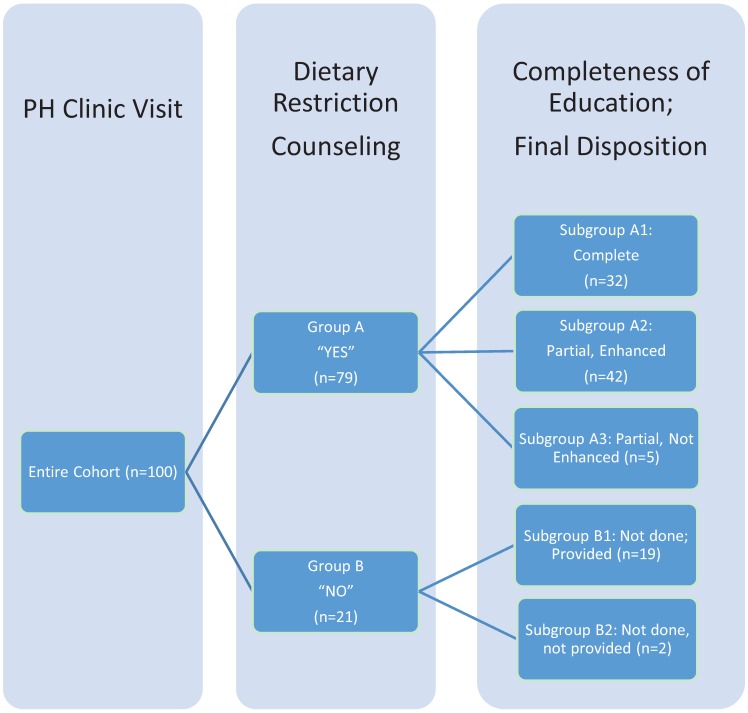
Breakdown of study cohort by previous education on sodium and fluid restriction and disposition in a pulmonary hypertension clinic. One hundred consecutive patients in Pulmonary Hypertension (PH) Clinic had PH and completed the sodium and fluid restriction questionnaire. Group A had either complete education (subgroup A1) or partial education (subgroups A2 and A3). Group B had no prior education or counseling. Subgroups A2 and B1 had education during the PH Clinic visit to complete the education (A2) or provide if there was none prior (B1). Subgroups A3 and B2 were missed opportunities to address the importance of sodium and fluid restriction and complete or provide the education required to do so.

As outlined in Table 1 and Figure 1, approximately one-third had received complete education and counseling with specific amounts of sodium and fluid restriction (subgroup A1). About half of the patients (*n* = 47) had received some counseling to restrict either fluid or sodium but no specific goals (subgroups A2 and A3). One-fifth (*n* = 21) had not received any instruction to restrict either fluid or sodium (subgroups B1 and B2).

Of the patients who had only received partial instruction (subgroups A2 and A3) or no instruction (subgroups B1 and B2), 44 (subgroup A2 and B1) received needed education during the PH Clinic visit and seven (subgroups A3 and B2) did not.

**Table 2:** Demographics of the entire cohort evaluated in the Pulmonary Hypertension Clinic.

Parameter	Entire Cohort	A1	A2	A3	B1	B2
Age (years)	63 ± 13	64 ± 12	63 ± 14	65 ± 14	64 ± 12	43 ± 33
Sex (% F)	70	72	71	100	63	0
NYHA FC	3 ± 1	3 ± 1	3 ± 1	3 ± 0	3 ± 1	3 ± 1
Stable (%)	85	94	86	60	74	100
Unstable (%)	15	6	14	40	26	0
Edema (%)	31	31	18	3	14	0
Diuretic [n (%)]	73 (73)	23 (72)	37 (88)	2 (40)	11 (58)	0 (0)
Diuretic Loop	46 (46)	15	24	1	6	0
Diuretic Loop+	19 (19)	6	10	1	2	0
Diuretic Spiron	5 (5)	2	2	0	1	0
Diuretic HCTZ	3 (3)	0	1	0	2	0
BNP (pg/mL)	302 ± 696	326 ± 383	521 ± 924	80 ± 53	377 ± 604	201 ± 263
6MWD (m)	337 ± 116	314 ± 106	346 ± 108	397 ± 98	343 ± 147	188 ± 0
RAP (mmHg)	8 ± 5	9 ± 5	8 ± 5	6 ± 2	8 ± 5	8 ± 4
MPAP (mmHg)	42 ± 13	42 ± 11	41 ± 14	32 ± 11	45 ± 16	42 ± 1

Key: Mean ± standard deviation; F = female; NYHA FC = New York Heart Association Functional Class; Stable = clinically stable; Unstable = clinically worse (typically more dyspnea); Diuretic = any diuretic on active medication list confirmed by clinician; Diuretic Loop = loop-inhibiting diuretic (e.g., furosemide or bumetanide); Diuretic Loop+ = loop-inhibiting diuretic plus metolazone or spironolactone; Diuretic Spiro = spironolactone only; Diuretic HCTZ = hydrochlorothiazide only; Edema = peripheral edema on physical examination; BNP = brain natriuretic peptide; 6MWD = six-minute walk distance in meters (m); MPAP = mean pulmonary artery pressure by echocardiogram; and RAP = right atrial pressure by echocardiogram.

## 4. Discussion

Sodium and fluid restriction is an important but perhaps underutilized strategy to manage volume overload in patients with heart failure. While challenging for the patient, instructions can be provided by the clinician in a brief period of time, particularly if educational materials are used to supplement the education. This process typically occurs in less than 5 min in the PH Clinic at MCF. Nonetheless, clinical experience seemed to indicate that patients often did not have prior counseling or had incomplete instructions. This study sought to quantify the frequency of missed opportunities to educate the patients in their prior medical evaluations and also during a current visit to the PH Clinic. To our knowledge, no prior study has specifically addressed sodium and fluid restriction education in patients with PH. Nonetheless, published guidelines recommend strategies to control volume overload as important general measures in PAH management [1,3,7].

Indeed, only 32% had received complete education in sodium and fluid restriction and 21% of patients had not even had such restriction mentioned to them prior to the PH Clinic visit. Most of the patients (68%) represented an opportunity for improvement in this regard and the majority received such instruction, though, unfortunately, 7% did not (subgroups A3 and B2). Whether the questionnaire on prior sodium and fluid restriction promoted the additional discussion and education that was provided during the PH Clinic visit is unknown but likely.

While the patients as a group were generally clinically stable, they were not at optimal goals of therapy as recommended by the WHO and American College of Chest Physicians guidelines [8,9], such as functional class II, BNP < 180 pg/mL, and 6MWD > 380 m. Echocardiogram revealed mildly elevated RAP and more severely abnormal MPAP. Optimization of therapy including dietary restriction of sodium and fluid was warranted. In addition, BNP is a surrogate marker of volume overload and right ventricular failure that correlates with survival in PAH [10] and can be lowered with sodium and fluid restriction and diuretic therapy. Interestingly, subgroup A3 seemed to be more at goal by minimal peripheral edema, BNP, 6MWD, and RAP than the other subgroups, although the number of patients was small. Perhaps this partially explains why additional instructions on sodium and fluid restriction were not provided during the PH Clinic visit, but this is speculative. Overall, most but not all patients were on diuretic therapy. Generally, loop-inhibiting diuretics, often spironolactone, were the treatment of choice with or without another class. Again, subgroup A3 was the least likely to be on diuretic therapy. Data was not collected on the questionnaire as to whether diuretic therapy was added at the PH Clinic visit, but the patients not on the therapy may represent an additional opportunity for improved treatment.

Whether this single center experience is representative of general practice is unknown and is a clear limitation of the study. The small number of patients in the subgroups warrants extreme caution in over-analyzing any differences that may be present. It should be noted that the data was collected prospectively but no study intervention was involved. All actions recorded regarding dietary counseling were at the discretion of the treating clinician. To that end, pathophysiological mechanisms in the management of sodium and water retention in this setting were not prospectively addressed. Notably, the control of each is renally managed by separate mechanisms involving the renin-angiotensin, aldosterone, and antidiuretic hormone signaling. The volume overload in heart failure results primarily from sodium retention with water retention occurring late in the course with attendant hyponatremia. In general, the clinical approach in this study was to seize the opportunity to emphasize dietary restriction of both sodium and fluid without attention to these mechanistic considerations.

In summary, guidelines often recommend sodium and fluid restriction as a complimentary strategy to manage right and left heart failure. The percentage with complete education in this regard was only 32% of the consecutive patient cohort seen in the PH Clinic at Mayo Clinic in Florida. Our questionnaire approach identified the remainder as an opportunity of improvement and perhaps promoted additional instruction.

## 5. Conclusions

Sodium and fluid restriction is an important but perhaps underutilized strategy to mitigate the clinical manifestations of right heart failure that often complicate pulmonary hypertension.
